# U. S. Department of Agriculture

**Published:** 1891-11

**Authors:** 


U. S. DEPARTMENT OF AGRICULTURE. Bureau of Animal Industry. Cause and Prevention of Swine Plague, by Theobold Smith, Ph.B., M. D. Twelve illustrations, colored. Government Printing Office.

The special report of the cause and prevention of swine plague, giving the result of experiments conducted under the direction of Dr. D. E. Salmon, Chief of the Bureau of Animal Industry, this is a sequel to the special report on Hog cholera. The author, after a resumé of prior investigations of swine plague in other countries, and in Illinois and Iowa, Maryland and New Jersey, gives the result of his experiments, and draws his practical deductions. So much has recently been published in this JOURNAL in support of and against Dr. Smith's conclusions that further review is unnecessary. Every veterinarian should carefully study the original, which he can obtain by application to the Congressman of his district.

